# Incremental Concurrent Model Synchronization using Triple Graph Grammars

**DOI:** 10.1007/978-3-030-45234-6_14

**Published:** 2020-03-13

**Authors:** Fernando Orejas, Elvira Pino, Marisa Navarro

**Affiliations:** 8 University of Paderborn, Paderborn, Germany; 9 ICREA, Open University of Catalonia, Barcelona, Spain; 10 Universitat Politècnica de Catalunya Barcelona, Barcelona, Spain; 11 Universidad del País Vasco, San Sebastián, Spain

## Abstract

In the context of software model-driven development, artifacts are specified by several models describing different aspects, e.g., different views, dynamic behavior, structure, distributed information, etc. Then, maintaining and repairing consistency of the whole specification are crucial issues if the models can be separately developed and updated. *Model Synchronization* is the process of restoring consistency after the update of one or several of the models. In the present work, we approach the case when conflicts may arise due to concurrently updating different models. Specifically, based on the *Triple Graph Grammar* approach, we propose an incremental algorithm $\mathtt{CSynch}$ for solving conflicts and repairing consistency. In addition, we identify and formalize when a synchronizing solution can be considered adequate and show that our procedure $\mathtt{CSynch}$ is sound and complete.

## 1 Introduction

In the context of model-driven development, artifacts are specified by several models describing different aspects, e.g., different views, dynamic behaviour, structure, interactions, etc. Moreover, a given set of models is said to be *consistent* if they describe some software artifact. Along the process of designing and implementing an artifact, and also after the artifact is implemented, it is common to modify or update some aspects of a given model, or of several models. These changes may cause inconsistencies between the given set of models. To restore consistency, we have to *propagate* these modifications to the rest of the models. This process is called *model synchronization*. If at each time, we just propagate the updates on one model, synchronization is said *sequential*, but if we propagate simultaneously updates on several models, synchronization is called *concurrent*. Most existing work on model synchronization deals with the sequential case, which is simpler than the concurrent one, since in the latter case we have to deal with possible inconsistencies between the modifications applied to different models, implying that in the synchronization process we may need to backtrack some updates. Moreover, the existing approaches to concurrent synchronization [37,38,14,11,34,35]

* This work has been partially supported by funds from the Spanish Research Agency (AEI) and the European Union (FEDER funds) under grant GRAMM (ref. TIN2017-86727-C2-1-R and TIN2017-86727-C2-2-R)

are based on sequentializing the process, i.e., on combining in some way propagation procedures defined in sequential synchronization. For this reason, these approaches are called propagation-based in [24], where it is shown that they have important limitations.

When the given concurrent updates are inconsistent among themselves, the synchronization procedure must backtrack some of these updates to restore consistency. However, in this case, not all synchronizing solutions are adequate. For instance, a possible inadequate solution could be backtracking all updates. None of the approaches considering conflict resolution [14,11,34,35] define any form of adequacy, other than consistency of the given result. Moreover, these approaches return only one possible solution, which may not coincide with the user wishes.

A simple but powerful way of describing a class of consistent (synchronized) models is by using a *Triple Graph Grammar* (TGG) [27,28], since this approach provides techniques and tools that allow the general formulation and resolution of problems associated with synchronization. In these years these techniques have had considerable success, producing a large number of contributions of proven utility.

In [10], it is claimed that synchronization procedures should be incremental, meaning that their execution cost should not depend on the size of the models, but on the size of the update, so that the final consistent models must not be rebuilt from scratch. Other approaches that propose incremental sequential synchronization procedures are [22,12,25]. In contrast, none of the existing approaches to concurrent synchronization is incremental.

The main contributions of this paper are:

- The definition of properties, other than consistency, to ensure the adequacy of concurrent synchronization solutions.
- The definition of a non-deterministic incremental algorithm for concurrent synchronization, that is not propagation-based, whose solutions satisfy our adequacy properties. The algorithm is nondeterministic to consider the possible choices of conflict resolution. In particular, the algorithm is shown to be complete, in the sense that it finds all adequate solutions to the synchronization problem.

The rest of the paper is organized as follows. In Sect. 2, we summarize the basic and preliminary notions and terminology required in the rest of the paper, and we introduce a running example. In Sect. 3 we introduce and formalize the properties that should be satisfied by the synchronizing solutions in order to be considered adequate. In Sect. 4, we propose our synchronizing algorithm which is proven to find all solutions that satisfy the properties mentioned above. Finally, in Sections 5 and 6 we present related work, conclude and describe future work.

## 2 Preliminaries

In this section, we describe some basic notions and terminology concerning model transformation and model synchronization by Triple Graph Grammars (TGGs). Moreover, we introduce the example that we will use in the paper.

## 2.1 Triple Graph Grammars

TGGs are a formalism developed by Schürr ([27]) to specify and implement model transformations. They are based on three main ideas:

- Models can be represented by some kind of graphs.
- Instead of representing a consistent pair of models by two graphs, it is better to do it by a *triple graph* ([27]) which, in addition, includes the correspondence between the elements of the two models.
- To specify the class of consistent triple graphs we use a (triple graph) grammar, i.e., a triple graph is *consistent* if it can be generated from a given start graph (typically, the empty graph) using the production rules of the grammar.

More precisely, a triple graph $\overline{G} = (G^S \overset{s_G}{\leftarrow} G^C \overset{t_G}{\rightarrow} G^T)$ consists of a *source graph* $G^S$ and a *target graph* $G^T$, which are related via the *correspondence graph* $G^C$ and two mappings (graph morphisms) $s_G : G^C \rightarrow G^S$ and $t_G : G^C \rightarrow G^T$ specifying how source elements correspond to target elements[3]. For simplicity, we use the notation $\langle G^S, G^T \rangle$ whenever the explicit correspondence graph can be omitted.

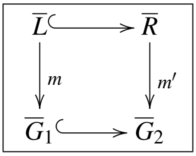

Then, a TGG $\mathcal{G}$ consists of a start triple graph[4], $\overline{SG}$, and a set of production rules of the form $r : \overline{L} \rightarrow \overline{R}$, where $\overline{L}$ and $\overline{R}$ are triple graphs and $\overline{L} \subseteq \overline{R}$. Then, $\mathcal{L}(\mathcal{G}) = \{\overline{G} \mid \overline{SG} \overset{*}{\Rightarrow} \overline{G}\}$ is called the class of *consistent models* and $\mathcal{D}(\mathcal{G}) = \{\overline{SG} \overset{*}{\Rightarrow} \overline{G}\}$ is the set of derivations defined by $\mathcal{G}$, where $\overset{*}{\Rightarrow}$ is the reflexive and transitive closure of the one step transformation relation $\Rightarrow$ defined as follows: $\overline{G}_1 \Rightarrow \overline{G}_2$ if there is a production rule $r : \overline{L} \rightarrow \overline{R}$ in $\mathcal{G}$ and a matching monomorphism $m : \overline{L} \rightarrow \overline{G}_1$ such that $\overline{G}_2$ can be obtained by replacing (the image of) $\overline{L}$ in $\overline{G}_1$ by (a corresponding image of) $\overline{R}$. Formally, this means that the diagram above on the right is a pushout in the category of triple graphs. In this case, we write $\overline{G}_1 \overset{r,m}{\Rightarrow} \overline{G}_2$, or just $\overline{G}_1 \Rightarrow \overline{G}_2$ if $r$ and $m$ are implicit.

For instance, in Fig. 1 we depict the graph grammar that we use as a running example to illustrate our techniques. It is a simplified, and slightly modified, version of the well-known transformation between class diagrams and relational schemas.

The graphs considered in this example are typed, which means that a *type graph* describes the different classes of nodes and edges of our triple graphs, in a similar way as a metamodel describes the kinds of elements that we have in a model. In particular, the type graph of our example is depicted on the left of Fig. 1. Source models, whose type graph is depicted on the left, consist of three kinds of nodes: classes, attributes and sub-attributes[5], and three kinds of edges: A (thick) edge between two classes represents a subclass relationship between them; attributes are bound to their associated classes and sub-attributes to their associated attribute, respectively, by the second and third kind of (thin) edges. Similarly, the type graph of target models is depicted on the right of the

[3] In the context of this paper, it does not make too much sense to speak about source and target models. Nevertheless, we have kept this terminology to simplify the notation for referring to each of the two models involved.

[4] As said above, without loss of generality, we consider that $\overline{SG}$ is always the empty triple graph.

[5] It is not necessary to associate any semantics to sub-attributes and sub-columns since we just use them to introduce a bit more complexity to the example.

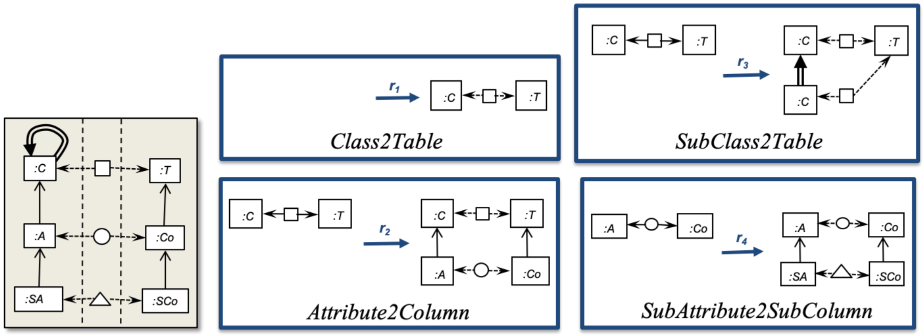

**Fig. 1.** Type graph, four rules for class-to-table transformations type triple graph, consisting of tables, columns and sub-columns, together with edges between them. Finally, in the middle, there is the type graph of the correspondence models, consisting of three kinds of nodes: square nodes to bind classes with their associated tables, round nodes to bind attributes with their associated columns, and triangle nodes to bind sub-attributes with their associated sub-columns.

The rules of the TGG defining the consistent transformations between class diagrams and relational schemas are depicted on the right of Fig. 1. Rule $r_1$, *Class2Table*, creates a new class and its corresponding table, together with the correspondence element that relates the class and the table. Rule $r_2$, *Attribute2Column*, given a class and a corresponding table, creates an attribute of that class, a related column of the table, and their associated correspondence element. Rule $r_3$, *Subclass2Table*, given a class and a corresponding table, creates a new subclass. In this case, the subclass is related to the table through a new correspondence element. Finally, rule $r_4$, *SubAttribute2SubColumn*, creates a new sub-attribute together with its corresponding sub-column.

On the left of Fig. 2 we depict a triple graph generated by this grammar. For instance, it could have been created from the empty graph, firstly, applying twice rule *Class2Table* to create classes $c_1$ and $c_2$ together with their associated tables $t_1$ and $t_2$ and correspondence elements; next, applying rule *Subclass2Table*, to create $c_3$ as a subclass of $c_2$, together with a correspondence element that specifies that $t_2$ is the table associated to $c_3$; finally, applying three times the rule *Attribute2Column*, to create attributes $a_1$, $a_2$ and $a_3$, together with their associated columns, the associated edges binding attributes and columns to their classes and tables, and their correspondence elements.

### 2.2 Model Update and Model Synchronization

For different reasons, given a consistent model $\overline{G}$, we may perform some modifications or updates in it producing a model $\overline{G}'$ that is not consistent anymore. Then the synchronization problem consists of repairing that model, so that it becomes consistent.

For instance, in our running example, we assume given the consistent model on the left of Fig. 2, and that two updates are defined on that consistent model: removing the subclass relation between $c_2$ and $c_3$ in the source model, and adding a new sub-column

$sco_3$ to the column $co_3$ in the target model. In the middle of the figure some elements of the triple graph have been marked. These marks ($\{+, \mathtt{x}, !, ?\}$) represent possible actions to be taken on the elements (adding, deleting or keeping them) as the result of the analysis performed in our algorithm, which we describe in the paper. Some elements have several marks that are contradictory. This tells us that some conflicting situations may arise when defining a repair. Finally, on the right of the figure, there is one possible repair of the marked triple graph that avoids conflicts and restores consistency. As we will see, this repair can be made incrementally, acting only on some elements (grey area) without having to rebuild the whole triple graph.

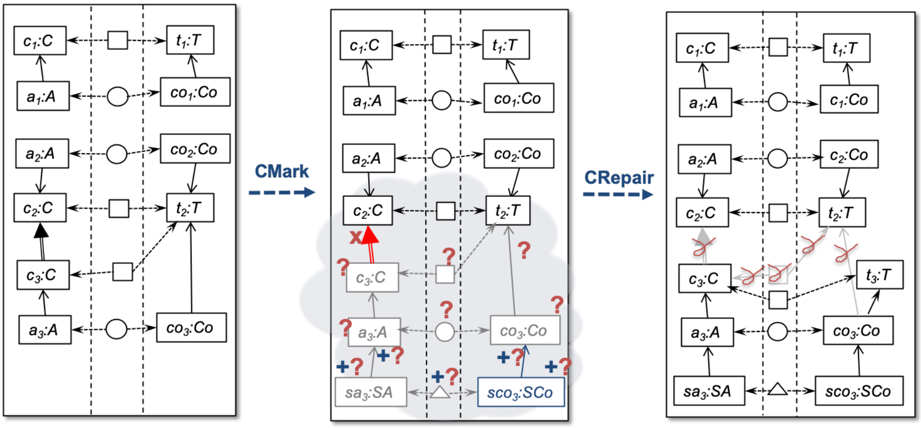

**Fig. 2.** Concurrent update, marked affected area with conflict and possible repair

Formally, an *update* or *modification* [8] $u$ on a graph $G$ is a span of inclusions $u\colon G \leftarrow K \rightarrow G'$ for some graph $K$. Intuitively, the elements in $G$ that are not in $K$ are the elements deleted by $u$, and the elements in $G'$ that are not in $K$ are the elements added by $u$. So, $K$ consists of all the elements in $G$ that remain invariant after the modification. When $K$ may remain implicit we will denote the update $u\colon G \leftarrow K \rightarrow G'$ by $u\colon G \Longrightarrow G'$.

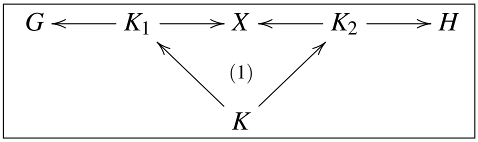

Updates can be composed and decomposed [24]. Given two updates $v\colon G \leftarrow K_1 \rightarrow X$ and $w\colon X \leftarrow K_2 \rightarrow H$, the *composition* of $v$ and $w$ is the update $u = w \circ v\colon G \leftarrow K \rightarrow H$ such that, roughly, $K$ is the intersection of $K_1$ and $K_2$, i.e. $K$ includes all the elements of $G$ that are neither deleted by $v$ nor by $w$. In addition, we say that $u$ *decomposes* into $v$ and $w$ if $u = w \circ v$ and moreover no element added by $v$ is deleted by $w$. Roughly this means that $X$ is the union of $K_1$ and $K_2$ with respect to the common part $K$. If $u$ decomposes into $v$ and $w$, we also say that $v$ is a *subupdate* of $u$, which we denote by $v \preceq u$, since in this case, $v$ adds and deletes less elements than $u$.

In the non-concurrent case, given a triple graph $\overline{G}$ and an update $w^S : G^S \Longrightarrow H^S$ on the source graph, the *synchronization problem* [16] is to find an update $w^T : G^T \Longrightarrow H^T$, such that $\overline{H}$ is consistent. In this case, we say that $w^T$ is the propagation of $w^S$.

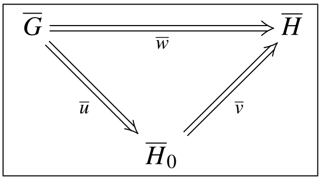

In contrast, in the concurrent case, given updates $u^S : G^S \Longrightarrow H_0^S$ and $u^T : G^T \Longrightarrow H_0^T$, or equivalently the triple graph update $\langle u^S, id, u^T\rangle : \overline{G} \Longrightarrow \overline{H}_0$, also called a *concurrent update*, the *concurrent synchronization problem* is to find a concurrent update $\overline{w} : \overline{G} \Longrightarrow \overline{H}$, such that $\overline{u} = \langle u^S, id, u^T\rangle$ is a subupdate of $\overline{w}$ and $\overline{H}$ is consistent. Previous work on this problem is based on building concurrent solutions by combining (in some way) $v^S$ and $v^T$, where $v^S$ (respectively, $v^T$) is the propagation of $u^T$ (respectively, of $u^S$). For this reason, in [24] these approaches are called *propagation-based*. However, as we pointed out in the introduction, in that paper it is shown that propagation-based approaches have important limitations.

A main problem in concurrent synchronization is that the given updates $u^S$ and $u^T$ may be *in conflict*. For instance, $u^S$ may delete a node $n$ in $G^S$ and $u^T$ may add an edge whose source-node is in correspondence to $n$ in $G^S$. When a concurrent update is in conflict it will be impossible to solve the synchronization problem, so we will have to backtrack (or to ignore) some of the deletions or additions in $\overline{u}$ to eliminate that conflict. In these situations, the concurrent synchronization problem needs to be reformulated. If $\overline{u}$ is in conflict, we would look for an update $\overline{w}$ such that a subupdate $\overline{u}'$ of $\overline{u}$ (i.e., some part of $\overline{u}$ not in conflict) is also a subupdate of $\overline{w}$. This is equivalent to saying that there is an update $\overline{v}$ such that $\overline{v} \circ \overline{u} = \overline{w}$, where $\overline{v}$ backtracks some conflicting updates included in $\overline{u}$. We must note that detecting conflicts is in general not an easy task, since $u^S$ and $u^T$ modify different models, so they do not directly interfere, which means that conflicts are never explicit. We may also note that, according to this definition, $\overline{id} : \overline{G} \Longrightarrow \overline{G}$, i.e., the identity modification that changes nothing, would always be a solution to the concurrent synchronization problem (in this case $\overline{v}$ would be the inverse of $\overline{u}$, so we would completely backtrack $\overline{u}$). Obviously, this is not the kind of solution that we want.

## 2.3 Dependency Relations

Incrementality of (sequential or concurrent) model synchronization requires two conditions for any given approach: to be able to identify what part of the given model is affected by an update, so that the rest can remain unchanged and we can concentrate on the affected part to build a solution; and that we can do this identification without having to fully analyze the given consistent model. Otherwise, the computational cost of a synchronization algorithm will always depend on the size of the given models.

Our approach to incrementality, which follows the ideas introduced in [25] for sequential synchronization, is based on the idea that the structure of a given consistent model depends essentially on the derivation that was used to create it. We mean that if we perform any update on the model, we just have to care about the parts of that derivation that are affected by the update. For instance, if the update consists of the deletion of some element, then the application of the rule that created that element in the original derivation and the further application of other rules that depend on that creation, will be considered the affected part of the derivation. It must be clear that this does not mean that, if $\overline{SG} \Rightarrow \overline{G}_1 \Rightarrow \cdots \Rightarrow \overline{G}_k \Rightarrow \cdots \Rightarrow \overline{G}$ is the derivation used to create $\overline{G}$, the deletion of an element in $\overline{G}_k$ will affect all the rule applications in the derivation $\overline{G}_k \Rightarrow \cdots \Rightarrow \overline{G}$, because some of these rule applications may be independent of that deletion. For instance, in our example, if the deleted element is a class, the creation of other classes, attributes or subattributes that are not related to that class would be independent of that deletion. Technically, the reason would be that the application of these rules is *sequentially independent* ([6]) of the application of the rule that created the class. In what follows we will denote by $d_{\overline{G}}$ the derivation[6] used to create $\overline{G}$.

Since in the synchronization algorithm we need to know which is the derivation used to create the given consistent model and storing and analyzing that derivation may be costly, the second idea of our approach is to define some dependency relations between the elements of $\overline{G}$ that allow us to know if the application of some rule depends on the application of another rule. We assume that these relations are stored together with $\overline{G}$. The first relation, called *strict dependency*, denoted $e_1 \lhd^{\overline{G}} e_2$, holds if $e_1$ is matched by the left-hand side of the rule that created $e_2$. For instance, in the triple graph on the left of Fig. 2, we have $c_2 \lhd^{\overline{G}} c_3$ and $t_2 \lhd^{\overline{G}} c_3$, since the application of rule *Subclass2Table* that creates $c_3$ has to match its left hand side to $c_2$ and $t_2$. The second relation, called *interdependency*, denoted $e_1 \bowtie^{\overline{G}} e_2$, holds if $e_1$ and $e_2$ are created by the same rule. For instance, in Fig. 2, $c_2 \bowtie^{\overline{G}} t_2$, since they are both created by the same application of the *Class2Table* rule in $d_{\overline{G}}$. Finally, *dependency*, denoted $\unlhd^{\overline{G}}$, is the reflexive and transitive closure of the union of $\lhd^{\overline{G}}$ and $\bowtie^{\overline{G}}$.

**Definition 1 (Dependency Relations [25]).** *Given a TGG $\mathcal{G}$ and a derivation $d_{\overline{G}}$: $\overline{SG} \stackrel{*}{\Rightarrow} \overline{G}$, we define the following relations on elements of $\overline{G}$:*

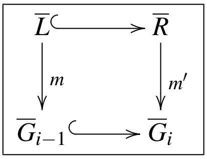

1. *Strict dependency: $\lhd^{\overline{G}}$ is the smallest relation satisfying that if $d_{\overline{G}}$ includes the transformation step depicted on the right, then for every $e$ in $\overline{L}$ and $e'$ in $\overline{R} \setminus \overline{L}$, $m(e) \lhd^{\overline{G}} m'(e')$.*
2. *Strict interdependency: $\bowtie^{\overline{G}}$ is the smallest relation satisfying that if $d_{\overline{G}}$ includes the transformation step depicted on the right, then for every $e, e'$ in $\overline{R} \setminus \overline{L}$, $m'(e) \bowtie^{\overline{G}} m'(e')$.*
3. *Dependency: $\unlhd^{\overline{G}} = (\lhd^{\overline{G}} \cup \bowtie^{\overline{G}})^*$.*

It may be noticed that there is a bijective correspondence between derivations (up to permutation equivalence) and their associated relations. This means that storing these relations together with a model is equivalent to storing the derivation used to create it.

## 3 Synchronizing Solutions for Concurrent Updates

According to what we discussed in the previous section, we consider the general problem of concurrent synchronization when there may be conflicts in the given concurrent update. Moreover, we assume that we are only interested in incremental solutions,

[6] It may be noted that there may be many derivations that lead to $\overline{G}$, here we assume that $d_{\overline{G}}$ is the one chosen to generate it.

which means that our solutions are assumed to preserve a certain triple subgraph of the given consistent model[7]. Finally, to avoid having to mention explicitly the TGG of the given synchronization problem, we will consider that we are working with a fixed TGG, $\mathcal{G}$, which has been given a priori.

**Definition 2 (Incremental Synchronizing Solutions).** *Given a concurrent update $\overline{u}$: $\overline{G} \Longrightarrow \overline{H}_0$, such that $\overline{G}$ is a consistent model, and given a submodel $\overline{G}_0 \subseteq \overline{G}$, a* concurrent incremental solution *of $\overline{u}$ with respect to $\overline{G}_0$ is an update $\overline{w}: \overline{G} \Longrightarrow \overline{H}$ such that $\overline{H}$ is consistent and $d_{\overline{H}}$ includes the derivation $d_{\overline{G}_0}$. Then, $SynchSol(\overline{G}, \overline{G}_0, \overline{u})$ is the set of all concurrent incremental solutions of $\overline{u}$ with respect to $\overline{G}_0$.*

In general, a concurrent synchronization problem may have several possible solutions especially if it has some conflicts, because in this case there may be different options of backtracking to eliminate the conflicts. To decide which solutions are "better", we may use different criteria but, unfortunately, these criteria may be contradictory. For this reason, we believe that it should be the user who decides which is the preferred solution. Nevertheless, there are solutions which may be considered inadequate or not fully adequate. For instance, backtracking all updates, so that the final outcome is the original consistent model, would technically be a correct solution, but we can not consider that it is adequate. The adequacy criteria that we consider are the following:

– *Maximal covering:* When $\overline{u}$ has conflicts, we would like that our solution backtracks as few as possible additions and deletions in $\overline{u}$, because users decided these additions and deletions. In this sense, the solution $\overline{w}$ has a maximal covering if $\overline{H}$ contains as many as possible elements that are added by $\overline{u}$ and as few as possible elements that are deleted by $\overline{u}$. In this case, a solution would be optimal if $\overline{H}$ includes all the elements added by $\overline{u}$ and no elements deleted by $\overline{u}$.
– *Minimal information loss:* The addition or deletion of an element in $\overline{u}$ may force the deletion of other elements from $\overline{G}$. Since these elements may include some information, their deletion will cause an information loss in the model, which we would like to minimize. In this sense, the solution $\overline{w}$ has minimal information loss if $\overline{H}$ cannot be extended to a solution that contains more elements from $\overline{G}$ without having more additions than $\overline{H}$.
– *Minimal unrelated additions:* The addition or deletion of an element in $\overline{u}$ may cause the addition of other elements in $\overline{w}$. For instance, if in our example we add a table the synchronization procedure will need to add its associated class. However, a solution may include other added elements that are not required by the given update. We consider that we should minimize this kind of additions.

**Definition 3 (Properties of Synchronizing Solutions).** *Given a derivation $d = \overline{SG} \overset{*}{\Rightarrow} \overline{G} \in \mathcal{D}(\mathcal{G})$ and a concurrent update $\overline{u}: \overline{G} \leftarrow \overline{K}_0 \rightarrow \overline{H}_0$, we say that a consistent incremental solution $\overline{w}: \overline{G} \leftarrow \overline{K} \rightarrow \overline{H} \in SynchSol(\overline{G}, G_0, \overline{u})$ has:*

1. Maximal covering: *if there does not exist any other solution $\overline{v} \in SynchSol(\overline{G}, G_0, \overline{u})$, such that $\overline{w}' \preceq \overline{v}'$, where $\overline{v}'$ is the largest common subupdate of $\overline{v}$ and $\overline{u}$, and $\overline{w}'$ is the*

[7] We may notice that if that subgraph is the empty graph then we would be looking for all possible solutions.

*largest common subupdate of $\overline{w}$ and $\overline{u}$, i.e., $\overline{v}'$ (resp. $\overline{w}'$) consists of all the additions and deletions that are both in $\overline{u}$ and $\overline{v}$ (resp. $\overline{w}$).*

2. Minimal information loss: *if there is no other update $\overline{v} \in SynchSol(\overline{G}, G_0, \overline{u})$, with $\overline{v} = \overline{G} \leftarrow \overline{K}' \rightarrow \overline{H}'$, such that $\overline{H} \overset{*}{\Rightarrow} \overline{H}'$, $\overline{K} \subset \overline{K'}$ and $(\overline{H}' \setminus \overline{K'}) = (\overline{H} \setminus \overline{K})$.*
3. Minimal unrelated additions: *if for any element $x \in \overline{H}$ added by $\overline{w}$, there is an element $y \in \overline{H} \cap \overline{G}$, such that $x \trianglelefteq^{H} y$.*

For instance, on the right of Fig. 2 we can see an example of a consistent solution which has maximal covering, minimal information loss and no unrelated additions. In contrast, in Fig. 3 neither the solution on the left nor the one in the middle have maximal covering, even though both of them are consistent. The solution on the right of Fig. 3 has maximal covering, minimal information loss, and no unrelated additions, but it is not comparable with the one in Fig. 2.

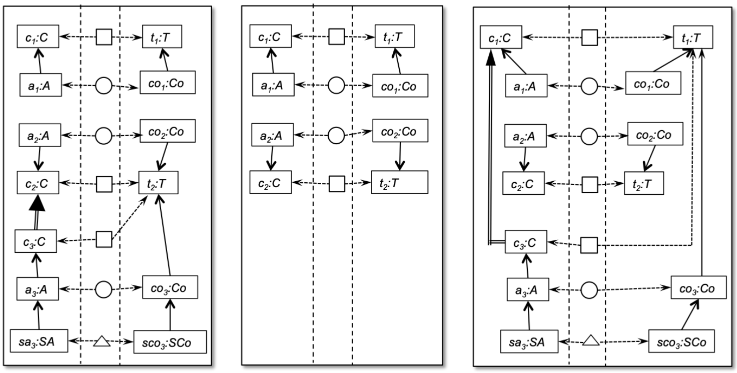

**Fig. 3.** Other three consistent solutions for example in Fig. 2.

## 4 An Incremental Procedure `CSynch`

In this section we propose a two-step nondeterministic incremental algorithm `CSynch` that allow us to find all solutions to the concurrent synchronization problem that are minimal, in the sense that they do not have unrelated additions, and that, moreover, have maximal covering and minimal information loss. More precisely, depending on the choices made we will get a different solution.

The algorithm is not based on propagation, but on using rules derived from the given TGG which allow us to identify which elements are affected by the update, to identify and solve possible conflicts, and to restore consistency. This identification is done by a marking algorithm `CMark` that simulates the addition and deletion of elements by applying these derived rules on the model such that some of its elements have being decorated with some marks from the set $\{+, \mathtt{x}, !, ?\}$. If an element $e$ is marked with any of these marks, it means that $e$ has been added or deleted by a user ($+$ or $\mathtt{x}$, respectively), it is required for an addition ($!$), or it is affected by a deletion ($?$). Technically, this means that every element of the model has an attribute called $\mathtt{marks} \subseteq \{+, \mathtt{x}, !, ?\}$ that denotes the set of marks that an element has at a given moment. Initially, before starting the synchronization process, it is assumed that $\mathtt{marks} = \emptyset$ for every element of the model. If it happens that, at certain point, an element is marked with different marks, it may denote an apparent conflict[8], which may need to be solved.

Since we need to know the dependencies between the marked elements, we will build extensions of the dependency relations of the given model $\overline{G}$. This extended relations are denoted $\trianglelefteq'$, $\triangleleft'$, and $\bowtie'$, i.e., $\trianglelefteq^{\overline{G}} \subseteq \trianglelefteq'$, $\triangleleft^{\overline{G}} \subseteq \triangleleft'$, and $\bowtie^{\overline{G}} \subseteq \bowtie'$. In addition, using these relations, CMark computes $\overline{G}_0$, the submodel of $\overline{G}$ not affected by the update.

Once the model is marked, an algorithm CRepair detects and solves conflicts and repairs the model. This process removes the marks of some elements and deletes the rest of them, in such a way that the final outcome is a consistent triple graph.

### 4.1 Marking

Before defining the marking algorithm that we use in the first step of our synchronization procedure, let us first explain how we deal with additions and deletions.

In our running example, let us suppose that the user has added an edge between the attribute $a_1$ and the class $c_2$ (perhaps to apply a refactoring to the given system). We know that in consistent models an edge between an attribute and a class is added when applying rule $r_2 : \overline{L} \to \overline{R}$, *Attribute2Column*. This rule, given a class and a table, adds to a given model an attribute, a column, edges between the attribute and the class, and between the column and the table, and a correspondence element that relates the attribute to the column. So, the idea is that in the synchronization algorithm, we are going to "simulate" the application of that rule to create the edge between $a_1$ and $c_2$, and to do this, we are going to apply a rule $r'_2 : \overline{L}' \to \overline{R}'$, derived from $r_2$, but before describing this rule, we have to take into account two questions:

1. Some of the elements that are created by $r_2$ may be already in the model. So, instead of creating them again, we include them in the left-hand side $\overline{L}'$ of the derived rule. Similarly, some other elements created by $r_2$ may coincide with other elements added by $\overline{u}$, then we will consider that $r'_2$ creates also these elements. For instance, if $\overline{u}$ would create an attribute $a$ and an edge associating $a$ to a class $c$, in this case, the associated marking rule would create simultaneously the attribute and the edge. But this is not enough to ensure that the final outcome is consistent, we need to be sure that all the elements in $\overline{L}'$ are in the final result. Otherwise, these elements could be deleted as a consequence of some other addition or deletion in the given update $\overline{u}$. For this reason, $r'_2$ will mark the elements in $\overline{L}' \setminus \overline{L}$ with $!$, expressing that they are required for the correctness of the result. In addition, the rule will also mark the elements created by the rule, i.e. in $\overline{R}' \setminus \overline{L}'$, with the mark $+$. This includes the elements added by $\overline{u}$, but also some other elements created by the rule. For

[8] As we will see, not all apparent conflicts are real conflicts.

instance, if we want to add the edge from $a_1$ to $c_2$ to the model, we must also add the edge from $co_1$ to $t_2$. Following these ideas, rule $r'_2$ is depicted on the left of Fig. 4. Moreover, on the right of that figure we also show which would be the associated marking rule, when we add an attribute to a class in a given model.

2. Since in the resulting model, $\overline{H}$, we assume that this edge from $a_1$ to $c_2$ is created using $r_2$, this means that we assume that in $\overline{H}$, the edge was created together with the attribute $a_1$, the corresponding column $co_1$, the edge between $a_1$ and $c_2$, the edge between $co_1$ and $t_2$, and the correspondence element between $a_1$ and $co_1$. However, in the original model $\overline{G}$ these elements were created using a different application of $r_2$, and together with them, some other elements were also created (in this case, the edges between $a_1$ and $c_1$ and between $co_1$ and $t_1$), that are still part of the model. So if we want $\overline{H}$ to be consistent, we will need to delete these elements from the model. As a consequence, we will mark them with ?, denoting that in principle, we have to delete them, and we will say that they have been *revoked*. Finally, if some elements are revoked, we may need to delete all the elements in the model that depend on them. So we will mark all these other elements with ?, expressing that they may need to be deleted too.

The case of marking the deletions in the update $\overline{u}$ is simpler. If an element $x$ in $\overline{G}$ is deleted by $\overline{u}$, we will just mark it with `x`, denoting that $x$ has to be deleted and, as before, we will mark all the elements that depend on $x$ with ?.

Finally, if we call $\overline{G}_0$ the graph consisting of all elements that have not been marked with $+$, `x`, or ? then, as a consequence of the way that the marking algorithm works, we can be sure that $\overline{G}_0$ is consistent (as shown in Thm. 1), since all elements in $\overline{G}_0$ were already in $\overline{G}$ and they are not dependent of any element that is not in $\overline{G}_0$. Hence, building our solution by adding to $\overline{G}_0$ some of the marked elements, using rules from the given TGG, ensures that the final result is consistent. Moreover, the algorithm would be incremental with respect to $\overline{G}_0$, since its elements will not be processed by `CRepair` (except for deleting some ! marks).

**Definition 4 (Derived Marking Rules).** *We say that a triple graph* $\overline{G}$ *is* decorated with marks *if each of its elements has a marking attribute* `marks` $\subseteq \{+, \mathtt{x}, !, ?\}$. *Let us denote as RemAttr*$(\overline{G})$ *the triple graph resulting from removing from* $\overline{G}$ *the attribute* `marks`.

*Given the rule* $r : \overline{L} \to \overline{R}$, *we say that* $r' : \overline{L}' \to \overline{R}'$ *is a* derived marking rule *from r for adding a set of elements X, if* $\overline{L}'$ *and* $\overline{R}'$ *are two decorated triple graphs such that:*

1. $\overline{L} \subseteq RemAttr(\overline{L}') \subseteq \overline{R}$, $RemAttr(\overline{R}') = \overline{R}$, *and* $X \subseteq \overline{R}' \setminus \overline{L}'$.
2. *All elements in* $RemAttr(\overline{L}') \setminus \overline{L}$ *are included in* $\overline{R}'$ *with the mark* !.
3. *All elements in* $\overline{R}' \setminus \overline{L}'$ *are included in* $\overline{R}'$ *with the mark* $+$.

For instance, the rule on the left of Fig. 4 is derived from the rule $r_2$ *Attribute2Column* to add a new arrow from an already existing attribute to an already existing class in the model. Notice that the elements that are really new, i.e., produced by the application of $r_2$, are marked with $+$, while the ones produced by $r_2$ but reused from the model by the derived rule, are marked with !. The rule on the right is also derived from *Attribute2Column* but now to add a new attribute to an existing class. As a consequence, there are not reused elements that should be marked with !.

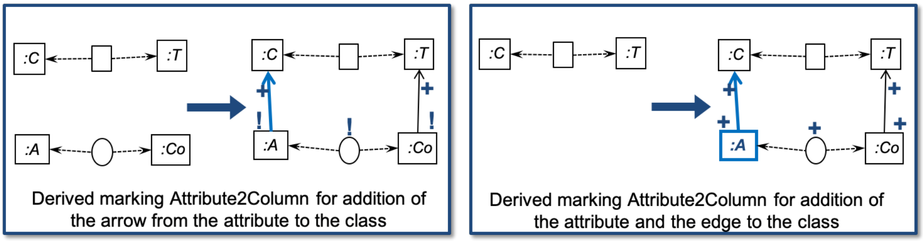

**Fig. 4.** Examples of derived marking rules

Now we can introduce the marking algorithm CMark following the explanations given above. $A$ and $D$ will be the set of elements that have to be added or deleted, respectively. Initially, we assume that $A$ and $D$ consist of the elements added and deleted by $\overline{u}$, and that the sets of marks are empty for all the elements in the model. Then:

**Algorithm 1** (CMark **Algorithm**)
*Initialize relations* $\trianglelefteq' = \trianglelefteq$, $\triangleleft' = \triangleleft$, *and* $\bowtie' = \bowtie$.

1. **Addition and revocation:** *For every element $x \in A$, select a marking rule $r' : \overline{L}' \to \overline{R}'$ derived from $r : \overline{L} \to \overline{R}$ that may be used to create $x$, and let $X \subseteq A$ be a set of elements that can also be created by $r'$:*
   - *Eliminate from A the elements in $\{x\} \cup X$.*
   - *Apply $r' : \overline{L}' \to \overline{R}'$.*
   - *Add ? to the attribute* marks *of every element which is not in $RemAttr(\overline{L}') \setminus \overline{L}$ but it is strictly interdependent with an element matched to $RemAttr(\overline{L}') \setminus \overline{L}$.*
   - *Add ? to the attribute* marks *of every element which is dependent on a ?-marked element.*
2. *Update the dependency relations adding the new dependencies and interdependencies defined by the application of the original rules used in 1. to relations $\trianglelefteq'$, $\triangleleft'$, and $\bowtie'$; and computing the new transitive closure.*
3. **Deletion:**
   - *Add* x *to the attribute* marks *of every element intended to be deleted.*
   - *Add ? to the attribute* marks *of every element that is dependent of an* x*-marked element.*
4. **Computing** $\overline{G}_0$: *Delete from $\trianglelefteq$, $\triangleleft$, and $\bowtie$ all elements marked with $+$, ?, or* x*. Then $\overline{G}_0$ would be the model generated by the derivation associated to the dependency relations.*

For instance, in the middle of Fig. 2 and Fig. 5 we can see examples of a marked model following the above algorithm. In the case of the example in Fig. 5, the concurrent update would consist of adding a subclass relation between classes $c_1$ and $c_2$, in the source; and, adding a new sub-column $sco_3$ to the column $co_2$ in the target. Again, in the model of the middle, some elements are marked with contradictory marks. Notice that now, possible conflicts arise because of trying to integrate concurrent additions. In fact, this example serves to illustrate that some additions may imply the deletion of elements created by the original derivation, for instance, it is the case of the table $t_2$. That is, some additions may imply revocation of original derivation steps.

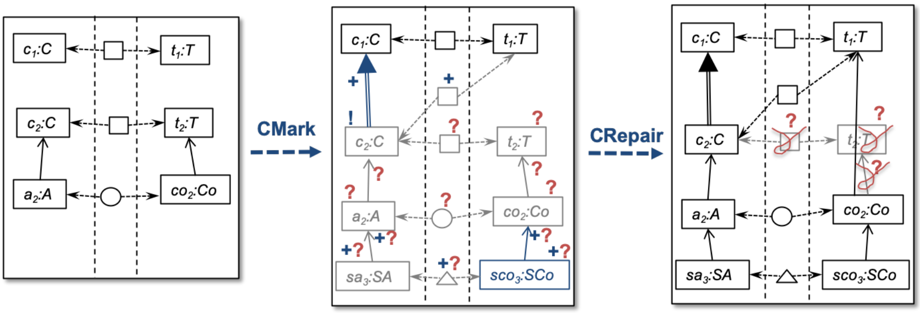

**Fig. 5.** Other example of concurrent updated, marked and possible repair

We must note that this algorithm is nondeterministic since, when we want to add an element $x$ to the model there may be more than one rule that can be used to create $x$. Then, choosing different rules will lead to different results of our synchronizing procedure.

### 4.2 Repairing and Conflict-Solving

The first idea underlying our repair algorithm, used as a second step of our synchronization procedure, is to extend the model $\overline{G}_0$, represented by the dependency relations $\trianglelefteq$, $\triangleleft$, and $\bowtie$, using rules from the given TGG, to include the elements that the user asked to add to the model (i.e. added by the given update $\overline{u}$) and to reduce the information loss. In particular, if in the marking process we decided to use a rule $r : \overline{L} \to \overline{R}$ to create an element $x$ required by $\overline{u}$ (i.e., to create $x$ we used a marking rule $r''$ associated to $r$), we will use another rule also derived from $r$, $r' : \overline{L}' \to \overline{R}$, where $\overline{L} \subseteq RemAttr(\overline{L}') = \overline{R}$ that unmarks all the elements that we marked with $+$ or $!$, i.e., if we remove all marks in $\overline{L}'$ we get $\overline{R}$. We call these rules *derived recreating rules*, because they create again (by reusing them) some elements that were originally in $\overline{G}$. We must note that, using the information in the dependence relations $\trianglelefteq'$, $\triangleleft'$, and $\bowtie'$, we may know which is the rule $r$. In particular, $\overline{L}$ would consist of $x$ and all the elements $y$ such that $x \bowtie' y$, and $\overline{R}$ would consist of all the elements $z$ such that $x \triangleleft' z$.

This idea for reusing elements from the original model has already been used in [12,25]. Notice that these rules eliminate the marks from the recreated elements, and as a consequence, the recreated elements will be now part of the solution.

The second idea for our repair algorithm is that we can also use derived recreation rules for reducing the information loss, including in the solution elements that were removed from the given model because they depended on elements that could have been deleted.

Finally, the third idea in which our algorithm is based is that, if we try to create an added element $x$ using the derived rule $r' : \overline{L}' \to \overline{R}$, if an element of $\overline{L}'$ is matched to an element $y$ of the model having the mark x, this means that we have discovered a conflict, because we have a conflict between the deletion of $y$ and the addition of $x$. As a consequence, we have two options, either we do not apply that rule, which is equivalent to backtrack the addition of $x$, or we do apply the rule, which would be equivalent to backtrack the deletion of the element including the mark x.

**Definition 5 (Derived Recreating Rules).** *Given a rule $r : \overline{L} \to \overline{R}$, we say that $r' : \overline{L}' \to \overline{R}$ is a* derived recreating rule[9] *from r if $\overline{L} \subseteq RemAttr(\overline{L}') = \overline{R}$, such that*

1. *The elements in $\overline{L}'$ from $\overline{L}$ must be matched to elements without marks.*
2. *The elements in $\overline{L}'$ not in $\overline{L}$ can be matched to elements with any mark.*

For instance, in Fig. 6 we can see some examples of some derived recreating rules.

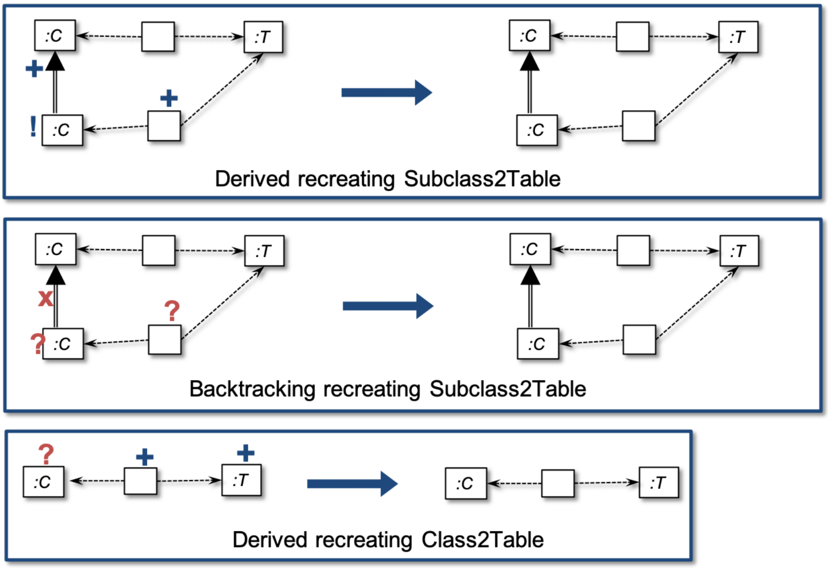

**Fig. 6.** Examples of derived recreating rules

[9] To be precise, recreating rules are like standard DPO rules, i.e. of the form $\overline{L}' \leftarrow \overline{R} \to \overline{R}$, which means that the rule does not add anything, since it just deletes the marks from the given elements. We may note that an unmarking rule is always applicable, since the gluing conditions always hold. For details, we may look at [17,15].

**Algorithm 2** (CRepair)

1. **Recreating and conflict solving**: *While there is a recreation rule that can be applied:*
   - *If there is an element marked* + *by a marking rule associated to a rule* $r:\overline{L}\rightarrow\overline{R}$ *and when trying to apply the associated recreating rule* $r':\overline{L}'\rightarrow\overline{R}$*, no element in* $\overline{L}'$ *is matched to an element including the* x *mark, then apply* $r'$ *and modify accordingly the dependency relations of the solution, adding to* $\lhd$ *and* $\bowtie$ *the dependency and interdependency relations between the elements matched by elements in* $\overline{L}$ *and* $\overline{R}$*; and computing the new transitive closure for* $\unlhd$.
   - *In the same situation as in the previous case, but where there is an element in* $\overline{L}'$ *that is matched to an element marked* x*, choose between applying* $r'$ *modifying accordingly the dependency relations of the solution, or replacing the mark* + *by the mark* x *for all elements matched by* $\overline{L}'$ *that include the mark* +.
   - *Otherwise, apply a recreating rule* $r':\overline{L}'\rightarrow\overline{R}$ *such that no element in* $\overline{L}'$ *is matched to an element marked* x *and modify accordingly the dependency relations of the solution.*
2. **Removing**: *Delete every marked element.*

That is, in step 1. of CRepair, we first try to recreate every + or ! element and to reduce information loss as much as possible. However, when we detect a conflict when trying to recreate an element marked +, it is nondeterministically chosen between applying the addition or the deletion. And in step 2 all elements still marked are removed from the model, because they needed to be deleted or because it was not possible to recreate them.

**Algorithm 3** (CSynch)

1. *Apply* CMark.
2. *Apply* CRepair

*The resulting update is* $\overline{w}:\overline{G}\Longrightarrow\overline{H}$*, where* $\overline{H}$ *is the result obtained by* CSynch.

### 4.3 Properties of CSynch

In this subsection we prove the properties that our algorithm satisfies. Firstly, we will prove that all solutions obtained by CSynch are consistent, incremental and they have no unrelated additions. We will also prove that CSynch can compute all incremental solutions that, in addition, have maximal covering and minimal information loss, provided that the right choices are made.

**Theorem 1.** *Given a consistent model* $\overline{G}$ *and an update* $\overline{u}:\overline{G}\Longrightarrow\overline{H}'$ *if the update* $\overline{w}:\overline{G}\Longrightarrow\overline{H}$ *is a solution obtained by* CSynch*, then:*

1. $\overline{H}$ *is consistent.*
2. $\overline{w}$ *is incremental with respect to the triple graph* $\overline{G}_0$ *computed by* CMark.
3. $\overline{w}$ *has no unrelated additions*
4. $\overline{w}$ *has minimal information loss.*

*Proof.* The last three properties are just a consequence of how CSynch is defined. Let us prove that $\overline{H}$ is consistent, but before we will prove that $\overline{G}_0$ is consistent.

Let $\overline{SG} \Rightarrow \overline{G}_1 \Rightarrow \cdots \Rightarrow \overline{G}_k \Rightarrow \cdots \Rightarrow \overline{G}$ be the derivation used to create $\overline{G}$ and let $\overline{SG} \Rightarrow \overline{G}_1 \Rightarrow \cdots \Rightarrow \overline{G}_i$ be its longest subderivation such that $\overline{G}_i \subseteq \overline{G}_0$, let us show that $\overline{G}_i = \overline{G}_0$, which implies the consistency of $\overline{G}_0$.

Suppose that there is an element $x \in \overline{G}_0$, which means that $x$ is not marked with $+$, ?, or x and it does not depend on any marked element, such that $x \notin \overline{G}_i$. Let $k$ be the earliest derivation step $\overline{G}_k \Rightarrow \overline{G}_{k+1}$, with $i < k$, where an element $x \in (\overline{G}_0 \backslash \overline{G}_i)$ was generated i.e., $x \in (\overline{G}_{k+1} \backslash \overline{G}_k)$. By definition of $\bowtie$, we know that $x \bowtie y$ for every $y \in \overline{G}_{k+1} \backslash \overline{G}_k$, and according to the definition of CMark, if $x$ has not any of those marks, then $y$ has not either. This means that $\overline{G}_{k+1} \backslash \overline{G}_k \subseteq \overline{G}_0$. Now, if $r : \overline{L} \rightarrow \overline{R}$ is the rule applied in the derivation step $\overline{G}_k \Rightarrow \overline{G}_{k+1}$ then there are two possibilities:

1. If the elements matched by $\overline{L}$ in $\overline{G}_k$ are already in $\overline{G}_i$, it would mean that this derivation step is sequentially independent from all derivation steps $\overline{G}_i \Rightarrow \cdots \Rightarrow \overline{G}_k$ and we would have $\overline{G}_i \overset{r}{\Rightarrow} \overline{G}_{i+1}$, with $\overline{G}_{i+1} \subseteq \overline{G}_0$, contradicting the hypothesis that $\overline{SG} \Rightarrow \overline{G}_1 \Rightarrow \cdots \Rightarrow \overline{G}_i$ was the longest subderivation such that $\overline{G}_i \subseteq \overline{G}_0$.
2. If the elements matched by $\overline{L}$ in $\overline{G}_k$ are not in $\overline{G}_i$, it would mean that $x$ depends on elements added in the derivation $\overline{G}_i \Rightarrow \cdots \Rightarrow \overline{G}_k$. Moreover, we know that all the elements $y$ generated in that derivation such that $y \trianglelefteq x$ are unmarked with $+$, ?, or x and therefore they are included in $\overline{G}_0$, because otherwise $x$ would not be in $\overline{G}_0$. But this contradicts the hypothesis that $x$ was an element in $\overline{G}_0 \backslash \overline{G}_i$ added in the earliest possible derivation step.

To prove that $\overline{H}$ is consistent, it is enough to notice that, because of how recreation rules are defined, if $\overline{G}_i$ is a consistent unmarked subgraph of a marked graph $\overline{G}'_i$, and $r' : \overline{L}' \rightarrow \overline{R}$ is a recreating rule associated to the rule $r : \overline{L} \rightarrow \overline{R}$, then if $\overline{G}'_i \overset{r'}{\Rightarrow} \overline{G}'_{i+1}$, we have that $\overline{G}_i \overset{r}{\Rightarrow} \overline{G}_{i+1}$ such that $\overline{G}_{i+1}$ is a consistent subgraph of $\overline{G}'_{i+1}$. In particular, if $\overline{G}'_0$ is the result obtained by applying the marking algorithm to $\overline{G}$ and $\overline{G}_0$ is its unmarked consistent subgraph, then applying the first step of CRepair leads to a sequence of recreation transformations $\overline{G}'_0 \overset{*}{\Rightarrow} \overline{G}'_k$. This means that given the associated sequence of transformations $\overline{G}_0 \overset{*}{\Rightarrow} \overline{G}_k$, we have that $\overline{G}_k$ is a consistent subgraph of $\overline{G}'_k$. Finally the second step of CRepair leads to $\overline{H} = \overline{G}_k$, which is the final result of CSynch. ■

Before showing the rest of the properties of CSynch, we must first define which is the subgraph $\overline{G}_0$ such any solution of CSynch is incremental with respect to it. In general, depending on the choices of CSynch, the consistent model $\overline{G}_0$ computed by CMark may be different, because the choice of rules used to add to $\overline{G}$ the elements added by $\overline{u}$ defines different markings. So, if we want that all solutions of CSynch are incremental over the same graph, we can take the intersection of all these $\overline{G}_0$.

**Definition 6 (Set of Computed Solutions).** *Given a consistent model $\overline{G} \in \mathcal{L}(\mathcal{G})$ and a concurrent update $\overline{u} : \overline{G} \leftarrow \overline{K} \rightarrow \overline{H}_0$, we denote by* CSynch$(\overline{G}, \overline{u})$ *the set of all possible solutions computed by the algorithm* CSynch *when $\overline{G}$ and $\overline{u}$ are given.*

*If for every $\overline{w} : \overline{G} \leftarrow \overline{K} \rightarrow \overline{H}$, we denote by $\overline{G}_w$ the subgraph of $\overline{G}$ computed by* CMark, *then we define $M(\overline{G}, \overline{u})$ as: $M(\overline{G}, \overline{u}) = \bigcap_{\overline{w} \in \mathtt{CSynch}(\overline{G}, \overline{u})} \overline{G}_w$.*

Obviously, if $\overline{w}$ is incremental over $\overline{G}_w$, then $\overline{w}$ is also incremental with respect to any submodel of $\overline{G}_w$.

**Proposition 1.** *If* $\overline{w} \in \texttt{CSynch}(\overline{G},\overline{u})$ *then* $\overline{w}$ *is incremental over* $M(\overline{G},\overline{u})$*.*

Finally, we can show that our algorithm is complete, i.e., that any consistent update that is incremental over $M(\overline{G},\overline{u})$, and satisfies the required properties, can be found by `CSynch`.

**Theorem 2.** *Given a consistent model* $\overline{G}$ *and an update* $\overline{u}:\overline{G} \Longrightarrow \overline{H}'$*, if* $\overline{w}:\overline{G} \leftarrow \overline{K} \rightarrow \overline{H}$ *is consistent, it has no unrelated additions, it has maximal covering and minimal information loss, and it is incremental over* $M(\overline{G},\overline{u})$ *then* $\overline{w} \in \texttt{CSynch}(\overline{G},\overline{u})$*.*

*Proof.* If $\overline{w}:\overline{G} \leftarrow \overline{K} \rightarrow \overline{H}$ is a consistent update, such that it has no unrelated additions, it has maximal covering and minimal information loss, and it is incremental over $M(\overline{G},\overline{u})$ this means that there is a derivation $d = \overline{SG} \Rightarrow \overline{H}_1 \Rightarrow \cdots \Rightarrow \overline{H}_k \Rightarrow \cdots \Rightarrow \overline{H}$ such that $\overline{M}(\overline{G},\overline{u}) \subseteq H_k$ for some $k$. Then, if we make the right choices in `CSynch` we will compute the solution $\overline{w}$. In particular, if `CMark` uses the same rule applications that are used in $d$ to generate the additions in $\overline{u}$, on the one hand, it will compute a model $\overline{G}_w$ that will be preserved by `CRepair` and that includes $\texttt{CSynch}(\overline{G},\overline{u})$. On the other hand, `CMark` will mark the model in such a way that if `CRepair` chooses the same rule applications (and in the same order) as in $d$, it will compute $\overline{H}$. ■

## 5 Related Work

The concurrent synchronization problem can be considered as a special case of the general problem of model (or graph) repair. In particular, in our case, a triple graph can be easily represented by a single graph, so the consistency problem for triple graphs can be seen as a special case of the consistency problem for graphs. The literature on model repair is quite large (see [23] for an excellent survey on this topic), so it does not make much sense to review all the existing approaches.

Concentrating on the problem of concurrent model synchronization, to our knowledge, the only works addressing the general problem[10] of concurrent synchronization are [38,14,11,24,34,35]. All these approaches are propagation-based, which means that synchronization is performed, first, propagating the updates in one model to the other model, then checking if there is any conflict between the propagated updates and the ones previously applied to that model and, if there are, solving the conflicts in some given way, and finally, propagating back the updates in the second model to the first one. That is, sequentializing concurrent synchronization. In all cases it is shown that the result obtained is correct, but no other properties are shown. In particular, in all these approaches, except in [38] the trivial solution obtained by backtracking all the updates would be considered a valid solution. On the other hand, the approach presented

[10] There is some work considering this problem in a more restrictive setting. For instance, in [26] models are restricted to tree-like structures and the target model is an abstract view of the source; and in [36] updates must be defined in terms of a given set of operations.

in [38] may be unable to find some existing solutions, as shown in [24]. Actually, that paper shows that propagation-based approaches have important limitations.

Our approach to incrementality is based on the ideas presented in [25], for the sequential synchronization case. Other approaches based on TGGs that propose incremental solutions to sequential model synchronization are [10,16,12,22] (and some variations on them) but all of them are, in our opinion, not completely satisfactory. In particular, even if the construction of the solution does not start from scratch but from the given consistent model $\overline{G}$, the approaches in [16,12,22] have to analyze the whole model $\overline{G}$ (for instance, to know what parts of $\overline{G}$ must be modified) so their cost depends on the size of the given model. This is not the case of [10], but their approach only works for the case when source and target models are bijective, which excludes the case where source models are views of target models (or vice versa). In addition, in [10,16,22] there may be information loss, which we avoid using the approach developed in [12] and also used in [25].

## 6 Conclusion

In this paper we have presented some properties that ensure the adequacy of solutions for a concurrent synchronization problem, together with an incremental non-deterministic algorithm that is able to return all possible sound solutions that, in addition, satisfy these properties.

Most existing algorithms for model synchronization return just one solution. We believe that this is not adequate, especially in the case of concurrent synchronization. In that context, one concrete solution corresponds to a specific way of solving the existing conflicts, which may not be the way that the user would have preferred. For this reason, we decided that completeness of the algorithm was an important issue. It is clear that, in practice, delivering to a user a relatively large set of solutions is not very convenient. However, we think that this is something to take into account at the implementation level, for instance, by showing conflicts in a stepwise way and, then, showing the different ways of solving each conflict.

From a theoretical viewpoint, our algorithm works for any kind of graphs. However, in practice, if the models have attributes, our algorithm would not be adequate. For example, let us suppose that we are working with a class of models where a certain attribute $a_1$ must be equal to the addition of attributes $a_2$ and $a_3$ and let us suppose that we are trying to synchronize a model $\overline{G}$, where $a1$ has some given value $v_1$, but $a_2$ and $a_3$ have no value, i.e., the synchronization algorithm should provide values to $a_2$ and $a_3$, such that their addition equals $v_1$. In this context, our algorithm would deliver infinite solutions, assigning to $a_2$ and $a_3$ all possible values $v_2$ and $v_3$ such that $v_1 = v_2 + v_3$. In general, dealing with attributed graphs in the context of sequential or concurrent model synchronization poses problems that are described in [1,21].

As future work, on the one hand, we plan to address the case of attributed models and, on the other hand, to extend our results to the multimodel case, i.e. when synchronizing more than two models. This case has specific complications, see, for instance [32,4]. It has already been approached in [34,35], but just as a straightforward generalization of [14], which means that it shares its limitations.
